# SUV3 helicase is required for correct processing of mitochondrial transcripts

**DOI:** 10.1093/nar/gkv692

**Published:** 2015-07-07

**Authors:** Paula Clemente, Aleksandra Pajak, Isabelle Laine, Rolf Wibom, Anna Wedell, Christoph Freyer, Anna Wredenberg

**Affiliations:** 1 Division of Metabolic Diseases, Department of Laboratory Medicine; Karolinska Institutet, Stockholm 17177, Sweden; 2 Center for Inherited Metabolic Diseases, Karolinska University Hospital, Stockholm 17176, Sweden; 3 Department of Molecular Medicine and Surgery, Science for Life Laboratory, Karolinska Institutet, Stockholm 17176, Sweden

## Abstract

Mitochondrial gene expression is largely regulated by post-transcriptional mechanisms that control the amount and translation of each mitochondrial mRNA. Despite its importance for mitochondrial function, the mechanisms and proteins involved in mRNA turnover are still not fully characterized. Studies in yeast and human cell lines have indicated that the mitochondrial helicase SUV3, together with the polynucleotide phosphorylase, PNPase, composes the mitochondrial degradosome. To further investigate the *in vivo* function of SUV3 we disrupted the homolog of SUV3 in *Drosophila melanogaster* (*Dm*). Loss of *dmsuv3* led to the accumulation of mitochondrial mRNAs, without increasing rRNA levels, *de novo* transcription or decay intermediates. Furthermore, we observed a severe decrease in mitochondrial tRNAs accompanied by an accumulation of unprocessed precursor transcripts. These processing defects lead to reduced mitochondrial translation and a severe respiratory chain complex deficiency, resulting in a pupal lethal phenotype. In summary, our results propose that SUV3 is predominantly required for the processing of mitochondrial polycistronic transcripts in metazoan and that this function is independent of PNPase.

## INTRODUCTION

All cellular systems require a variety of regulatory mechanisms to remove redundant or incorrect RNAs. Although clearly defined for cytosolic RNAs into different decay pathways, mitochondrial RNA degradation is more elusive with several protein complexes proposed to function as mitochondrial degradosomes (1). Mammalian mitochondrial DNA (mtDNA) is a small circular multi-copy genome, encoding for 22 tRNAs, 11 mRNAs and 2 rRNAs. All genes are transcribed as long precursor RNAs from two main promoters within the mitochondrial matrix (2). The mitochondrial tRNAs are interspersed throughout the genome and the tRNA punctuation model (3,4) predicts that these primary transcripts undergo endonucleolytic cleavage between gene boundaries in a coordinated fashion. Thus, a range of different RNAs species, including mRNAs, rRNAs and tRNAs, as well as a variety of non-coding RNAs and antisense RNAs, are generated and a putative degradation machinery has to distinguish between them, to ensure controlled gene expression. tRNAs are modified after excision from the polycistronic transcripts, by a 3′ CCA addition as well as base modifications for correct folding and stability (5,6), while messenger RNAs are post-transcriptionally polyadenylated, with the exception of ND6 in human cells (7) and mouse (8). In bacteria, chloroplasts and some eukaryota polyadenylation is proposed to promote degradation, while in most eukaryotic cytosolic mRNAs polyadenylation promotes export to the cytosol, stability and translation (9). In contrast, the role of polyadenylation of mitochondrial transcripts is less clear, and despite its requirement for the formation of functional stop codons, its role in RNA stability and translation is unknown (10). For instance, although polyadenylation has been proposed to be required for correct translation of the majority of transcripts (11), not all mitochondrial mRNAs are polyadenylated and polyadenylation-like signals have even been observed as part of the degradation pathway (12). Thus, characterization of the mitochondrial degradosomes might shed light on mechanisms regulating mitochondrial RNA stability.

In a variety of different biological systems the minimal degradosome for RNA degradation comprises an exonuclease and a helicase (13). Recently, several components of the mitochondrial RNA processing machinery as well as factors involved in translation and degradation pathways have been localized to specific foci, termed mitochondrial RNA granules (14–17). For instance, the ATP-dependent 3′-5′ RNA/DNA helicase, suppressor of *var1* (SUV3) was first discovered in yeast as suppressor of the *var1* deletion phenotype, a component of the mitochondrial small ribosomal subunit (18). Deletion of *var1* results in a translational defect, and the rescue by SUV3 was proposed to be due to increased mitochondrial transcript availability (19). SUV3 belongs to a highly conserved Ski2 family of DExH-box RNA helicases, with orthologs found in eukaryotes to *Rhodobacter* (20). Yeast SUV3 was later shown to interact with the ribonuclease Dss1 to form the mitochondrial degradosome (21,22). Dss1 is absent in higher eukaryotes, attributable to the lack of polyadenylation of yeast mitochondrial RNA, suggesting that SUV3 either adopted a new function, or interacts with a different ribonuclease.

Silencing of the human *SUV3* gene product (SUPV3L1) or the expression of a dominant-negative variant in human cells results in increased mRNA steady-state levels, as well as the accumulation of mRNA decay intermediates, processing byproducts and antisense RNAs (23). In agreement with its role in degradation, SUV3 was later shown to interact with the mitochondrial polynucleotide phosphorylase, PNPase, to form the mammalian mitochondrial degradosome (24). In contrast, SUV3 and PNPase do not functionally co-localize in an RNAi screen for mitochondrial RNA processing genes (25). Silencing of PNPase itself resulted in conflicting results (26), and PNPase has been proposed to both degrade and extend 3′ tails *in vitro*, as well as localize to the intermembrane space (27), where it is involved in the import of RNAs (28). Additionally, although SUV3 has been shown to unwind DNA and RNA duplexes as well as RNA/DNA heteroduplexes it seems to have an increased affinity to DNA (29,30).

The mitochondrial genome of *Drosophila melanogaster* (*Dm*) and mammals is conserved, encoding the same genes, differing in gene order and gene expression patterns (31,32). Additionally, *Dm* mtDNA contains a large AT-rich non-coding region of unknown function, instead of a displacement loop, and multiple transcription sites have been proposed in the fly. Nevertheless, many regulators of mitochondrial gene expression have been shown to be conserved (33–39), and we therefore decided to use *Dm* as a model system to investigate the role of SUV3 in mitochondrial gene expression. Knockdown of DmSUV3 by RNAi, resulted in a severe mitochondrial dysfunction and pupal lethality. As predicted by its role in RNA maintenance, mitochondrial mRNA and anti-sense RNA steady-state levels were increased, while rRNA steady-state levels were not affected. In contrast, several tRNAs were severely reduced, and we observed the accumulation of processing intermediates, suggesting that SUV3 activity is important for the processing and maturation of mitochondrial transcripts *in vivo*.

## MATERIALS AND METHODS

### *Drosophila* stocks and maintenance

For *in vivo* knockdown of *dmsuv3* a w;UAS-*dmsuv3-*RNAi; line was obtained from the National Institute of Genetics (NIG-Fly, Japan) (#9791R-2). For *in vivo* knockdown of *dmpnpase* a w;UAS-*dmpnpase*-RNAi; line was obtained from Vienna Drosophila Resource Center (VDRC) (#108198). Ubiquitous knockdown of *dmsuv3* or *dmpnpase* was achieved by crossing the UAS-RNAi lines to the driver line *daughterless*GAL4 (w;;daGAL4). The stock carrying the P-element insertion in *dmsuv3*, w;;P{EPgy2}CG9791^EY12505^/TM6B-GFP, was obtained from Bloomington Drosophila Stock Center (#20354). All fly stocks were backcrossed for at least 6 generations into the white Dahomey background (w^Dah^). All fly lines were maintained at 25°C and 60% humidity on a 12h:12h light:dark cycle on a standard yeast–sugar–agar medium.

### Constructs

Full-length *dmsuv3* cDNA was obtained from the Drosophila Genomics Resource Center (LD23445). A single G deletion in the cDNA in position 849 was corrected by site-directed mutagenesis, using Phusion High Fidelity DNA Polymerase (Finnzymes). The cDNA was PCR amplified and cloned in the pEGFP-N3 plasmid (Clontech) to generate a *dmsuv3*-GFP fusion construct. The *dmsuv3*-GFP fusion construct was subsequently cloned in pAc5.1/V5-His A plasmid (Life Technologies) for expression in Schneider 2R+ cells. Primers used for the cloning of DmSUV3 are listed in Supplementary Table S1.

### Cell culture and transfection

HeLa cells were cultured in high-glucose DMEM (Life Technologies) supplemented with 10% fetal bovine serum at 37°C in a 5% CO_2_ atmosphere. Schneider 2R+ cells were cultured in Schneider's *Drosophila* Medium (Life Technologies) supplemented with 10% fetal bovine serum at 25°C. For co-localization studies, HeLa cells or Schneider 2R+ cells were transfected using Lipofectamine 3000 (Life Technologies) or a calcium phosphate transfection kit (Sigma-Aldrich), following the manufacturer's instructions. 48h after transfection cells were stained with 50 nM Mitotracker Red CMXRos (Life Technologies) and fixed (only HeLa cells) with 4% PFA. Images were obtained on a Nikon Confocal Microscope.

### Hatching rates

For adult hatching rate measurements, eggs were collected during a 4h time window and transferred to vials (100 eggs/vial) to ensure standard larval density. Hatching of adult flies was monitored in regular intervals.

### Biochemical evaluation of respiratory chain function

Isolation of mitochondria from third-instar larvae was performed as previously described (33) with some modifications in buffer composition. Third-instar larvae were washed and homogenized in 250 mM sucrose, 2 mM EGTA and 5 mM Tris pH 7.4 with 1% BSA. Protein concentration of the mitochondrial preparations was determined using a Qubit fluorometer and mitochondria were resuspended in 250 mM sucrose, 15 mM K_2_HPO_4_, 2 mM MgAc_2_, 0.5 mM EDTA and 0.5 g/l BSA, pH 7.2 for determination of the activities of the respiratory chain complexes. Biochemical activities of respiratory chain complexes were determined as previously described (40).

### Blue Native polyacrylamide gel electrophoresis (BN-PAGE) and in-gel activity assays

BN-PAGE and in-gel staining for complex I and IV activities was performed as previously described (33). In brief, mitochondria were pelleted and lysed in 1% digitonin, 0.1 mM EDTA, 50 mM NaCl, 10% glycerol, 1 mM PMSF and 20 mM Tris pH 7.4 for 15 min on ice. After removing insolubilized material by centrifugation, samples were loaded on 4–10% polyacrylamide gradient gels. In-gel complex I activity was determined by incubating the BN-PAGE gels in 2 mM Tris–HCl pH 7.4, 0.1 mg/ml NADH and 2.5 mg/ml iodonitrotetrazolium chloride. In-gel complex IV activity was determined by incubating the BN-PAGE gels in 50 mM phosphate buffer pH 7.4, 0.5 mg/ml 3.3′-diamidobenzidine tetrahydrochloride (DAB), 1 mg/ml cytochrome *c*, 0.2 M sucrose and 20 μg/ml catalase. Staining was performed at room temperature.

### DNA isolation, qPCR and Southern blot analysis

Genomic DNA from third instar larvae was isolated with the DNeasy Blood and Tissue Kit (Qiagen), following manufacturer's instructions. For Southern Blot experiments, 1 μg of each DNA sample was digested with XhoI to linearize mtDNA molecules and precipitated, followed by separation on a 0.8% agarose gel and blotted to Hybond-N+ membranes (GE Healthcare). Membranes were hybridized with [^32^P]-labeled double stranded DNA probes and exposed to PhosphorImager screens. dsDNA probes were labeled with [^32^P] dCTP (PerkinElmer), following the PrimeIt II kit (Stratagene). qPCR quantification of mtDNA levels was performed in triplicates using 5 ng of DNA and Platinum SYBR Green qPCR supermix-UDG (Life Technologies). Primers used in qPCR experiments are listed in Supplementary Table S1.

### RNA isolation and quantitative RT-PCR (qRT-PCR)

Total RNA was isolated, using the ToTALLY RNA kit (Ambion, Life Technologies) and quantified with a Qubit fluorometer (Life Technologies) unless stated otherwise. Reverse transcription was performed using High Capacity cDNA Reverse Transcription Kit (Applied Biosystems, Life Technologies). qRT-PCR was performed on a ViiA 7 system (Life Technologies), using the TaqMan Universal Master Mix II, with UNG and TaqMan assays (Life Technologies) to quantify mitochondrial mRNAs or Platinum SYBR Green qPCR supermix-UDG (Life Technologies) to quantify the transcripts containing tRNA-mRNA junctions. TaqMan assays and primers used for qPCR are listed in Supplementary Table S1.

### Northern blot analysis of mitochondrial RNAs

Steady-state levels of mitochondrial transcripts were determined by northern blot analysis, using 3 μg of total RNA, essentially as previously described (33,34). Mitochondrial tRNAs and processing intermediates were separated by neutral 10% PAGE or 1% MOPS-formaldehyde agarose gels. The aminoacylation status of tRNAs was determined on TRIZOL-isolated (Invitrogen) RNA, separated by acidic-UREA 6.5% PAGE. Separated RNAs were transferred to Hybond-N+ membranes (GE Healthcare) and hybridized with either randomly [^32^P]-labeled dsDNA probes, [^32^P]-labeled strand-specific RNA probes or with strand-specific [^32^P]-end labeled oligonucleotide probes. Primers used to generate dsDNA probes have been previously described (33,34). Primers used as oligonucleotide probes are listed in Supplementary Table S1.

### *In organello* transcription and translation assays

Mitochondria were isolated from third instar larvae and *in organello* transcription assays were performed as previously described (34). In brief, 200 μg of fresh mitochondria were incubated for 45 min in transcription buffer (30 μCi [^32^P]-UTP, 25 mM sucrose, 75 mM sorbitol, 100 mM KCl, 10 mM K_2_HPO_4_, 50 μM EDTA, 5 mM MgCl_2_, 1 mM ADP, 10 mM glutamate, 2.5 mM malate, 10 mM Tris- HCl pH 7.4 and 5% (w/v) BSA), followed by RNA extraction, separation on a 1% formaldehyde agarose gel and transferring to Hybond-N+ membranes (GE Healthcare). Mitochondrial *de novo* translation in isolated mitochondria was assayed as previously described (34), using EXPRESS protein labeling mix easy-tag (Perkin Elmer). Equal amounts of total mitochondrial protein were separated on 15% SDS-PAGE gels, followed by staining with 1 g/l Coomassie Brilliant Blue in a 20% ethanol, 10% acetic acid solution. Gels were then destained, dried and exposed to a PhosphorImager screen to visualize the mitochondrial translation products.

### RNA circularization and RT-PCR

RNA circularization and RT-PCR was performed as previously described (41). In brief, mitochondrial RNA extracts were treated with TURBO DNase (Ambion, Life Technologies) to remove contaminant DNA. 10 ng of mitochondrial RNA were circularized with T4 RNA ligase (New England Biolabs), precipitated and cDNA synthesis was performed, using gene-specific primers with the GeneAmp RNA PCR kit (Applied Biosystems, Life Technologies). PCR products were cloned into pCRII-TOPO (Life Technologies) following manufacturer's instructions and direct sequencing of the plasmid from single colonies was performed as previously described (42). Primer sequences for RT-PCR and subsequent PCR to amplify the region containing the poly(A) tails are listed in Supplementary Table S1.

### Statistical analysis

All data are presented as mean ± standard error of the mean (SEM). Unpaired *t*-test was used to analyze the statistical significance of the results.

## RESULTS

### DmSUV3 is a mitochondrial protein

**Figure 1.**
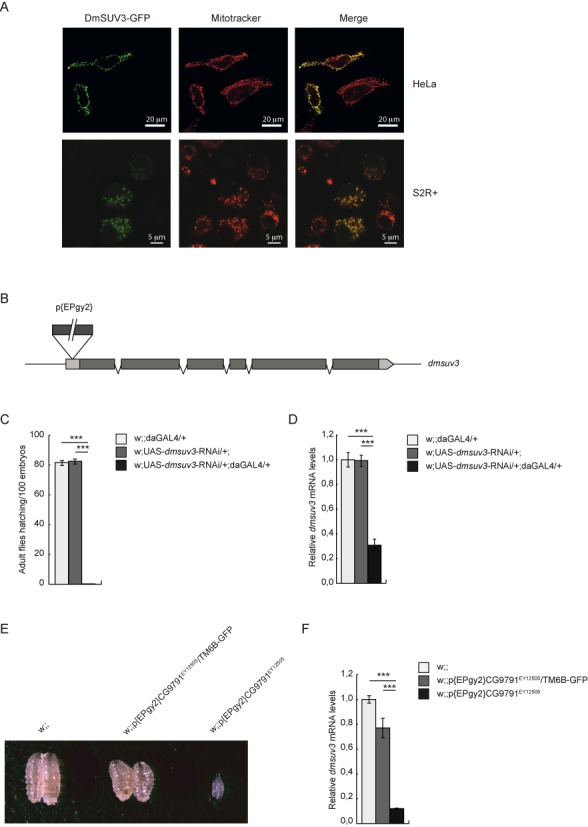
DmSUV3 is a mitochondrial protein essential for *Drosophila melanogaster* development. (**A**) HeLa and Schneider 2R+ cells expressing a GFP-tagged DmSUV3 fusion protein (DmSUV3-GFP) stained with mitotracker red CMXRos to visualize the mitochondrial network. Scale bars represent 20 μm (top panel) or 5 μm (bottom panel). (**B**) Schematic representation of the P-element insertion in the *dmsuv3* gene. (**C**) Hatching rates in *dmsuv3* knock down (w;UAS-*dmsuv3*-RNAi/+;daGAL4/+) and control (w;UAS-*dmsuv3*-RNAi/+; and w;;daGAL4/+) lines. (**D**) qRT-PCR of *dmsuv3* transcript levels in knock down (KD) and control larvae at 5 days after egg laying (ael). RP49 transcript was used as endogenous control. All data are represented as mean ± SEM (***P < 0.001, n = 7). (**E**) Body size comparison in control (w;;), heterozygous P-element insertion (w;;P{EPgy2}CG9791^EY12505^/TM6B-GFP) and homozygous P-element insertion (w;;P{EPgy2}CG9791^EY12505^) larvae at 3 days ael. (**F**) qRT-PCR of *dmsuv3* transcript levels in control, heterozygous P-element insertion and homozygous P-element insertion larvae at 3 days ael. RP49 transcript was used as endogenous control. All data are represented as mean ± SEM (*P < 0.05, **P < 0.01, ***P < 0.001, *n* = 5).

*Dm* has been proven as an excellent model system to study post-transcriptional regulation mechanisms *in vivo*, with many factors involved in mitochondrial gene expression conserved between *Dm* and humans. Although SUPV3L1 was proposed to have a homolog in *Dm* (20), functional validation has not yet been performed. A standard BLAST search for the SUPV3L1 ortholog in *Dm* identified a single candidate, encoded by the gene CG9791, sharing a 55% identity on the protein level (Supplementary Figure S1). Mitochondrial targeting prediction suggests that DmSUV3 is a mitochondrial protein using either Mitoprot II (0.969) or Target P (0.946) software. Additionally, transient expression of a GFP-tagged DmSUV3 construct in HeLa and *Drosophila* Schneider 2R+ cells confirmed a mitochondrial co-localization of the DmSUV3-GFP fusion protein (Figure 1A). Surprisingly, we observed no nuclear localization.

### *Dmsuv3* silencing is lethal in *Drosophila melanogaster*

In order to analyze the *in vivo* function of DmSUV3 we disrupted *dmsuv3* expression in two different approaches. First, we ubiquitously silenced *dmsuv3* expression by RNAi knockdown (KD), exploiting the UAS-GAL4 system (see materials and methods). Our second independent fly model carries a P-element transposon (w;;P{EPgy2}CG9791^EY12505^/TM6B-GFP) insertion in the 5′ untranslated region (UTR) of *dmsuv3* (Figure 1B).

Induction of *dmsuv3* silencing in the KD line resulted in a delay in larvae development and lethality during the pupal stage (Figure 1C). Silencing of *dmsuv3* expression levels was confirmed by qRT-PCR, with a severe reduction in *dmsuv3* steady-state levels to 30% in KD larvae compared to control lines (Figure 1D). Flies heterozygous for the P-element insertion are viable and fertile, with an ∼20% reduction in *dmsuv3* mRNA levels (Figure 1E and F). In contrast, the homozygous disruption of DmSUV3 by P-element insertion resulted in larval lethality by four days after egg laying (ael) (Figure 1E), with expression levels of *dmsuv3* corresponding to only 10% of control samples (Figure 1F). Thus, the disruption of DmSUV3 resulted in a dose-dependent lethality, suggesting that DmSUV3 is essential for fly development.

### DmSUV3 deficiency leads to a severe respiratory chain dysfunction

**Figure 2.**
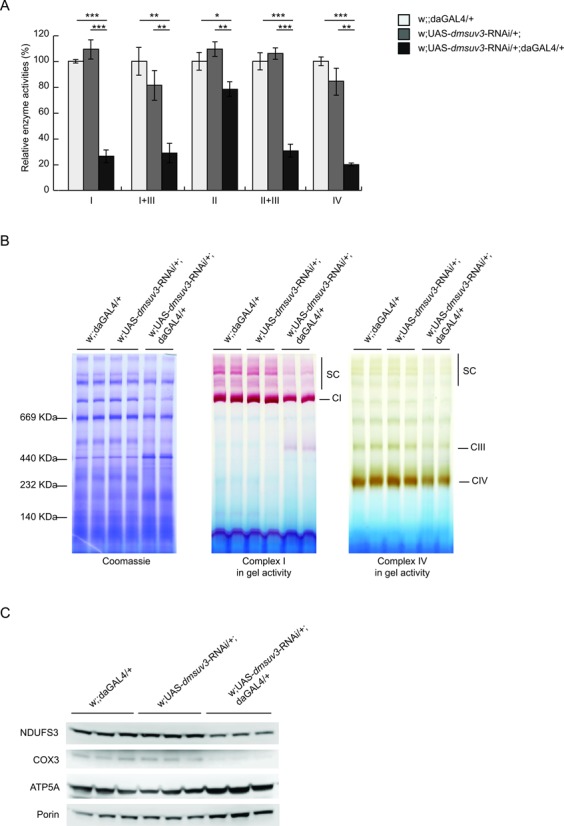
DmSUV3 deficiency leads to a severe respiratory chain dysfunction. (**A**) Relative enzyme activities of respiratory chain enzyme complex I (NADH coenzyme Q reductase), complex I+III (NADH cytochrome *c* reductase), complex II (succinate dehydrogenase), complex II+III (succinate cytochrome *c* reductase) and complex IV (cytochrome *c* oxidase) in mitochondrial protein extracts from *dmsuv3* KD (w;UAS-*dmsuv3*-RNAi/+;daGAL4/+) and control (w;UAS-*dmsuv3*-RNAi/+; and w;;daGAL4/+) larvae at 5 days ael. Data are represented as mean ± SEM (*P < 0.05, **P < 0.01, ***P < 0.001, *n* = 5). (**B**) BN-PAGE analysis and in-gel staining of complex I and complex IV activities in mitochondrial protein extracts from control and *dmsuv3* KD larvae at 5 days ael (CI, complex I; CIII, complex III; CIV, complex IV; SC, supercomplexes). (**C**) Western blot analysis of peptide steady-state levels of mitochondrial-encoded COX3 and nuclear-encoded NDUFS3 and ATP5A respiratory chain subunits in mitochondrial protein extracts from control and *dmsuv3* KD larvae at 5 days ael. Porin was used as a loading control.

To investigate the biochemical consequences of *dmsuv3* knockdown in mitochondria, we measured the activity of the respiratory chain complexes in isolated mitochondria from KD larvae at 5 days ael. As shown in Figure 2A, silenced larvae present with a severe decrease of complexes I, I+III, II+III and IV activities to ∼20–30% of control samples. In contrast, the exclusively nuclear encoded complex II was only mildly affected (∼80% of control samples) in the *dmsuv3* deficient larvae (Figure 2A), suggesting that the respiratory chain defect is of mitochondrial origin. Similar OXPHOS defects have previously been shown in two independent fly models with mitochondrial dysfunction (33,34), resulting in reduced cellular ATP/ADP ratios (33). A mitochondrial-derived defect was further confirmed by Blue Native polyacrylamide gel electrophoresis (BN-PAGE) followed by in-gel staining for complexes I and IV activities (Figure 2B). In agreement with the biochemical measurements, silenced larvae presented with decreased activities of fully assembled complex I and complex IV at 5 days ael (Figure 2B). Furthermore, western blot analysis revealed a decrease of the mitochondrial-encoded subunit COX3 and the nuclear-encoded subunit NDUFS3 (Figure 2C), supporting that KD larva present with reduced levels of respiratory chain complexes. Together these results indicate that DmSUV3 is required for mitochondrial function in *Drosophila*.

### DmSUV3 deficiency leads to increased mitochondrial mRNA but not rRNA stability

**Figure 3.**
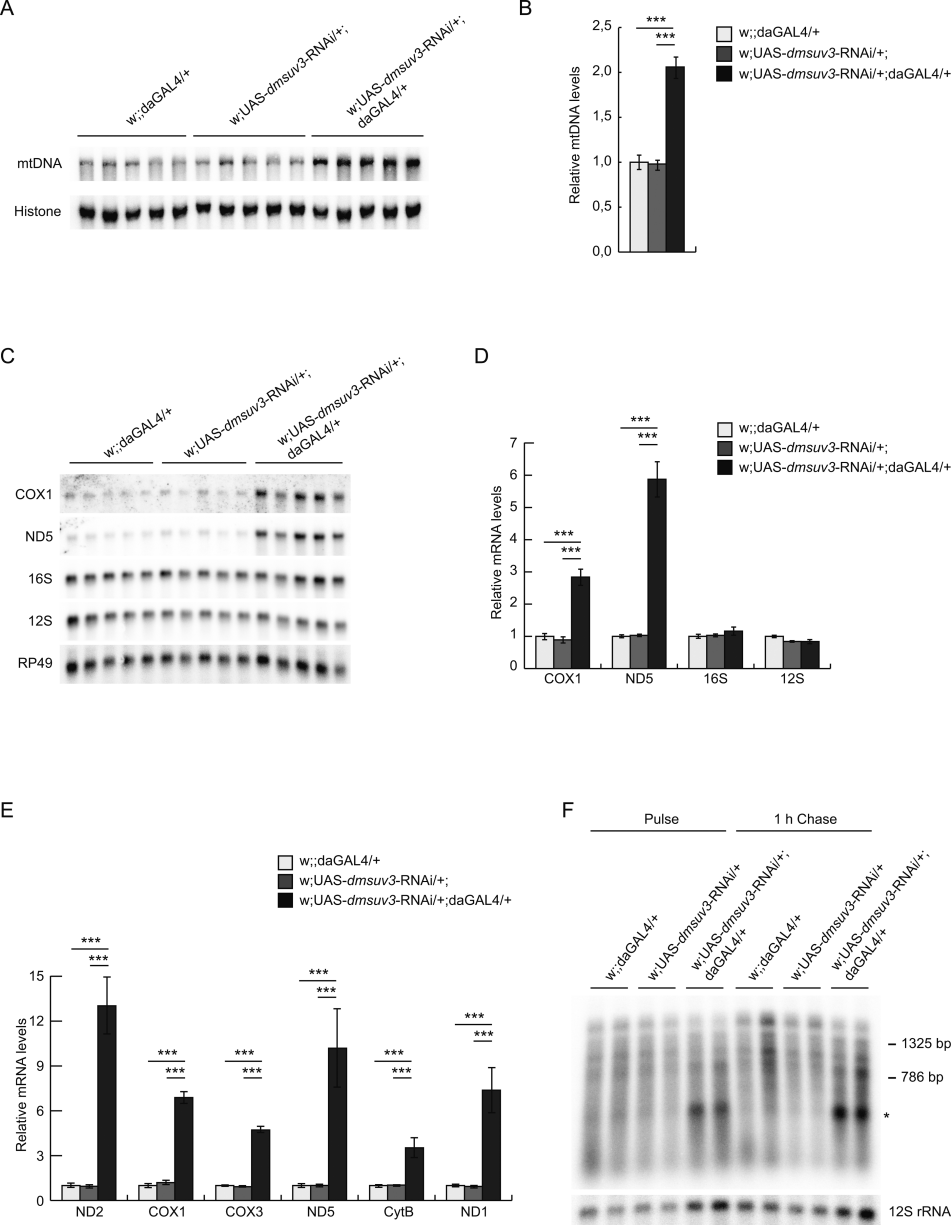
DmSUV3 deficiency leads to increased mRNA stability. (**A**) Southern blot analysis of mtDNA levels in *dmsuv3* KD (w;UAS-*dmsuv3*-RNAi/+;daGAL4/+) and control (w;UAS-*dmsuv3*-RNAi/+; and w;;daGAL4/+) larvae at 5 days ael. (**B**) Quantification of mtDNA steady-state levels in control and *dmsuv3* KD larvae at 5 days ael. Loading of gels was normalized to nuclear DNA levels detected with a probe against histone. (**C**) Northern blot analysis of the steady-state levels of mitochondrial mRNAs and rRNAs in KD and control larvae at 5 days ael. (**D**) Quantification of mitochondrial mRNA and rRNA steady-state levels in control and *dmsuv3* KD larvae at 5 days ael. Loading of gels was normalized to the transcript encoding the nuclear ribosomal protein 49. (**E**) qRT-PCR of mitochondrial mRNAs in KD and control larvae at 5 days ael. RP49 transcript was used as endogenous control. (**F**) *De novo* mitochondrial transcription in isolated mitochondria from control and *dmsuv3* KD larvae at 5 days ael. Mitochondrial rRNA 12S was used as a loading control. All data are represented as mean ± SEM. (*P < 0.05, **P < 0.01, ***P < 0.001, *n* = 5).

To further investigate the effects of *dmsuv3* silencing on mitochondrial function, we assessed mtDNA levels by qPCR or Southern blot analysis. Although P-element insertion had no effect on mtDNA steady-state levels in larvae 3 days ael (Supplementary Figure S2A), reduced *dmsuv3* expression in 5-day-old larvae lead to increased mtDNA steady-state levels (Figure 3A and B). We therefore conclude that this might be a compensatory response in mitochondrial biogenesis as previously described both in fly (33,34) and mouse (43,44) models with defects in mitochondrial gene expression.

SUV3 has been proposed to be involved in RNA degradation and we therefore proceeded to analyze the steady-state levels of mitochondrial transcripts, using qRT-PCR and northern blot analyses. Mitochondrial mRNA steady-state levels were significantly increased in both *dmsuv3-*silenced (Figure 3C–E) and P-element insertion (Supplementary Figure S2B) larvae. Surprisingly, northern blot analysis failed to identify the accumulation of any obvious RNA degradation products for any of the transcripts analyzed (Supplementary Figure S2C), in contrast to previously reported results in human cell lines (23). Additionally, although mRNA steady-state levels were increased, the ribosomal RNAs 12S and 16S were unaffected in the KD larvae (Figure 3C, D), suggesting DmSUV3 is not involved in post-transcriptional regulation of the mitochondrial rRNAs. Interestingly, the increase in mRNA steady-state levels coincided with only a mild effect on *de novo* transcription in KD larvae 5 days ael, which stabilized during a 1h chase (Figure 3F). Although we cannot exclude that differential intra-mitochondrial ribonucleotide pools might affect transcription in KD larvae, our results suggest that the loss of DmSUV3 leads to a specific stabilization of mitochondrial messenger RNAs. Additionally, we observed a profound synthesis of a single transcript of unknown function (Figure 3F, asterisk). We were unable to determine the identity or function of this transcript, but the low levels of this transcript in control samples suggests that this transcript is either rapidly turned over in controls or the loss of DmSUV3 results in increased synthesis.

### DmSUV3 deficiency decreases mitochondrial tRNA steady-state levels and impairs mitochondrial translation

**Figure 4.**
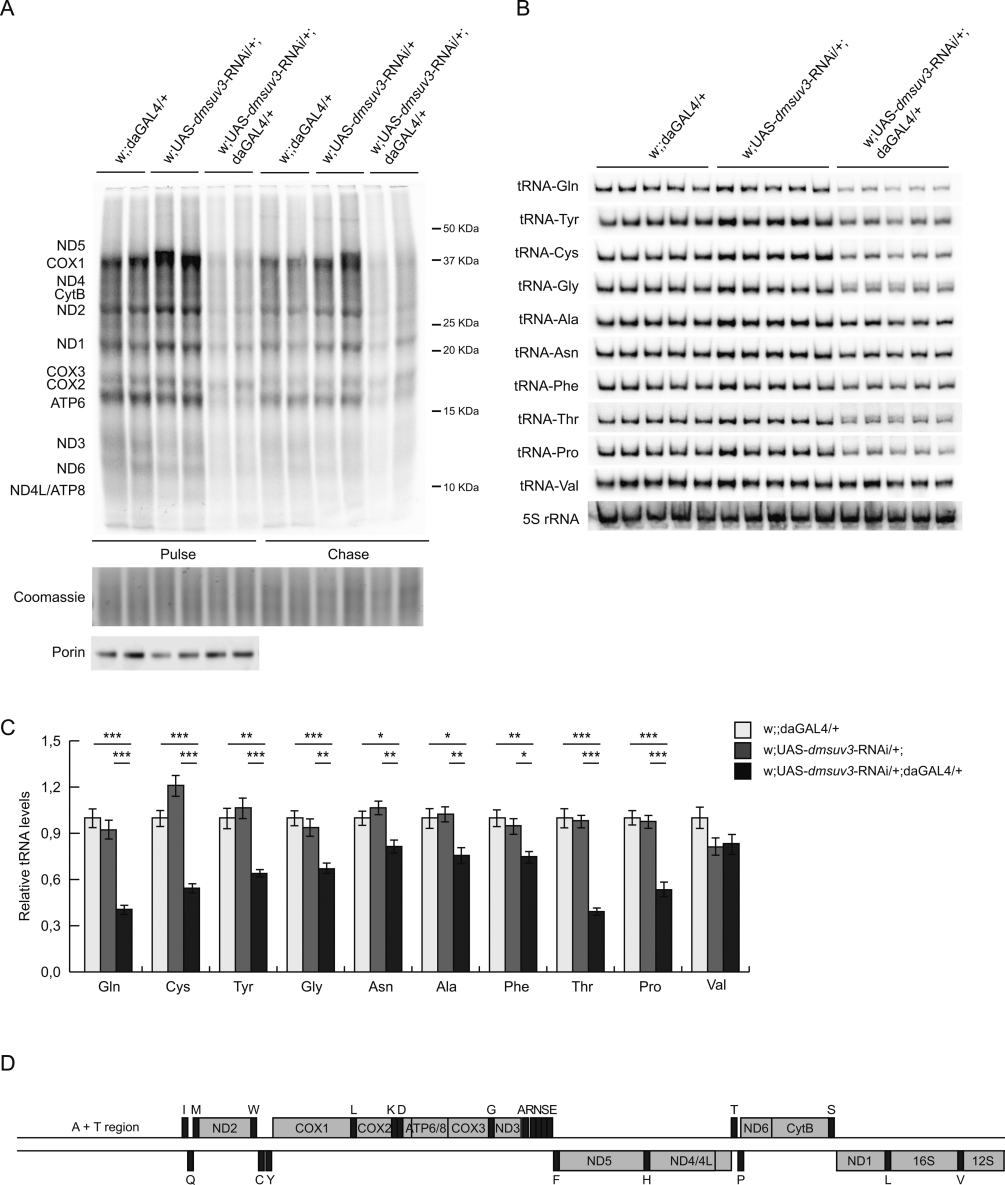
*dmsuv3* deficiency decreases mitochondrial tRNA steady-state levels and impairs mitochondrial translation. (**A**) *De novo* mitochondrial translation in isolated mitochondria from *dmsuv3* KD (w;UAS-*dmsuv3*-RNAi/+;daGAL4/+) and control (w;UAS-*dmsuv3*-RNAi/+; and w;;daGAL4/+) larvae at 5 days ael. Loading was normalized to the total mitochondrial protein content per μl of sample. Coomassie Blue staining of the gel and porin Western blot of the input samples are shown as loading controls. (**B**) Northern blot analysis of the steady-state levels of mitochondrial tRNAs in KD and control larvae at 5 days ael. (**C**) Quantification of mitochondrial tRNA steady-state levels in control and KD larvae at 5 days ael. Loading of gels was normalized to 5S ribosomal RNA. (**D**) Schematic linearized map of *D. melanogaster* mtDNA.

The increased steady-state levels of mRNAs *per se* do not explain the severe biochemical phenotype observed in *dmsuv3* KD larvae, and we therefore performed *in organello* labeling of mitochondrial translation products in isolated mitochondria from larvae at 5 days ael. As shown in Figure 4A, *dmsuv3-*silenced larvae show a generalized decrease in the synthesis of all mitochondrial-encoded polypeptides, consistent with the decreased mitochondrial subunit steady-state levels (Figure 2C).

To investigate how decreased degradation of mitochondrial mRNAs can lead to a translational defect, we measured mitochondrial tRNA steady-state levels by northern blot analysis. To our surprise the majority of the tested tRNAs (9 out of 10) were significantly decreased in KD larvae 5 days ael (Figure 4B and C). Interestingly, the most severely affected tRNAs were consistently flanked by non-coding sequences, such as tRNA^Gln^, tRNA^Cys^, tRNA^Pro^ or tRNA^Thr^ (Figure 4B-D). This observation was independent of the strand or the position within the genome.

### Loss of DmSUV3 leads to altered processing of mitochondrial transcripts

Our results thus far suggest that the steady-state level of various mitochondrial RNA species is differentially regulated, and that increased mRNA levels, in combination with decreased tRNA levels, can result in a severe respiratory chain defect. However, as many of the mRNAs and tRNAs are encoded on the same polycistronic precursor RNA we hypothesised that post-transcriptional processing or maturation of the RNAs might be affected.

**Figure 5.**
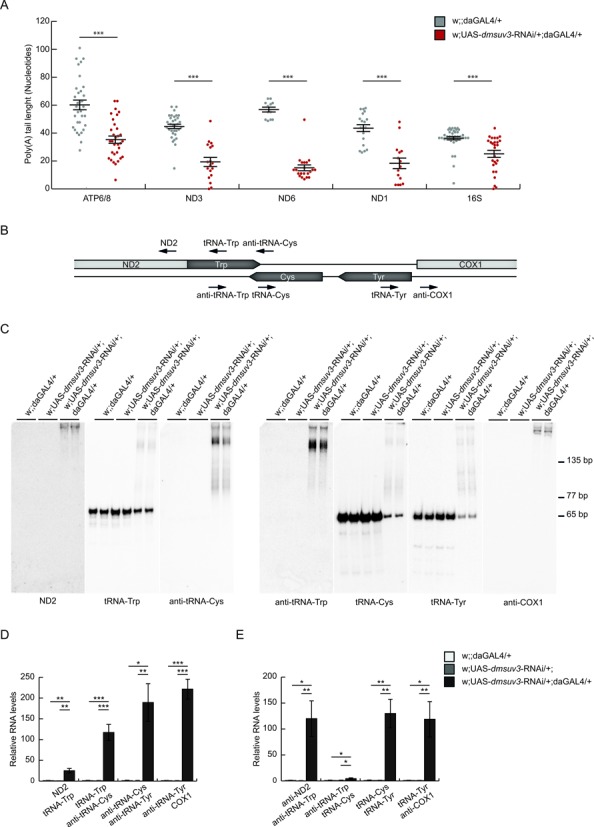
*dmsuv3* deficiency leads to a reduction of poly(A) tails and altered processing of mitochondrial tRNAs. (**A**) Poly(A) tail length in individually sequenced clones after transcript circularization in *dmsuv3* KD (w;UAS-*dmsuv3*-RNAi/+;daGAL4/+) and control (w;;daGAL4/+) larvae at 5 days ael. Mean poly(A) tail length varied between 34 and 60 adenines in control (gray; *n* ≥ 15) and 11 and 35 adenines in KD (red; *n* ≥ 24) samples. Data are represented as mean ± SEM (***P < 0.001). (**B**) Schematic representation of the end-labeled oligonucleotide probes (black arrows) used in northern blot experiments. (**C**) Northern blot experiments on PAGE separated total RNA using oligonucleotide probes, detailed in (**B**), against tRNA^Trp^ (left panel), tRNA^Cys^ and tRNA^Tyr^ (right panel), and their 5′ and 3′ flanking regions in *dmsuv3* KD (w;UAS-*dmsuv3*-RNAi/+;daGAL4/+) and control (w;UAS-*dmsuv3*-RNAi/+; and w;;daGAL4/+) larvae at 5 days ael. (**D**) qRT-PCR of the mitochondrial precursor transcripts containing ND2, tRNA^Trp^, anti-tRNA^Cys^ and COX1 in KD and control larvae at 5 days ael. RP49 transcript was used as endogenous control. (**E**) qRT-PCR of the mitochondrial precursor transcripts containing tRNA^Cys^ and tRNA^Tyr^ in KD and control larvae at 5 days ael. RP49 transcript was used as endogenous control. All data are represented as mean ± SEM (*P < 0.05, **P < 0.01, ***P < 0.001, *n* = 5).

We thus investigated the polyadenylation status of mitochondrial RNAs, as we previously demonstrated that altered polyadenylation in flies due to the loss of the leucine-rich pentatricopeptide repeat containing (LRPPRC) protein homolog, the bicoid stability factor (BSF), can result in a severe respiratory chain defect (33). Surprisingly, by cloning and sequencing we observed a significant decrease in poly(A) tail length in several mtDNA-encoded transcripts, including ATP6/8 (decreased to 60%), ND3 (33%), ND6 (23%), ND1 (38%), as well as 16S rRNA (60%) (Figure 5A).

We further performed high-resolution northern blot analysis to investigate tRNA integrity. While aminoacylation of tRNAs was not affected (Supplementary Figure S3A), we observed aberrant processing intermediates of a subset of mitochondrial tRNAs (Figure 5B and C). Strikingly, tRNAs with the most severely affected steady-state levels also presented with unprocessed intermediates. In order to determine the nature of these transcripts, we performed detailed mapping, using end-labeled oligonucleotides covering sequences flanking either side of the affected tRNAs as well as sequences on the tRNA and its complement strand (Figure 5B). We consistently observed unprocessed transcripts of tRNAs flanked by noncoding sequence (e.g. tRNA^Tyr^, tRNA^Cys^, tRNA^Gln^) in knockdown (Figure 5B, C and Supplementary Figure S4A) and also in P-element larvae (Supplementary Figure S3B), despite not showing an overall decrease in tRNA steady-state levels. In contrast, tRNAs with coding sequence directly adjacent were unaffected (e.g. tRNA^Val^, Supplementary Figure S4B). Overall, tRNAs were found attached to their 3′ flanking (e.g. tRNA^Trp^, Figure 5C), 5′ flanking RNA sequences (tRNA^Gln^, Supplementary Figure S4A) or 3′ and 5′ flanking sequences (tRNA^Tyr^, Figure 5C) suggesting that processing of both tRNA ends is affected in the absence of DmSUV3. These unprocessed extensions ranged from just a few nucleotides (e.g. tRNA^Gly^, Supplementary Figure S4B) to entire antisense tRNAs (e.g. tRNA^Trp^, Figure 5C). We observed only one exception to this. Analysis of the downstream region of tRNA^Phe^ did not reveal any incorrectly processed tRNA despite being flanked by non-coding DNA on its 3′ end (Supplementary Figure S4B).

To further confirm the accumulation of precursor RNA molecules containing tRNAs, we performed strand-specific qRT-PCR spanning the tRNA junctions, previously analysed by northern blot analysis. We found a significant accumulation of the 5′ and 3′ junctions of tRNA^Trp^, tRNA^Cys^ and tRNA^Tyr^ (Figure 5D and E) and the 3′ junction of tRNA^Gln^ (Supplementary Figure S4D) in KD larvae, supporting the observation that precursor transcripts accumulate in the absence of DmSUV3. In agreement with the northern blot results, we found no accumulation of COX3-tRNA^Gly^ or tRNA^Gly^-ND3 junctions in KD larvae (Supplementary Figure S4E) in comparison to w;;daGAL4/+ control, as the unprocessed extension of this tRNA is too short to be amplified with our qRT-PCR analysis.

**Figure 6.**
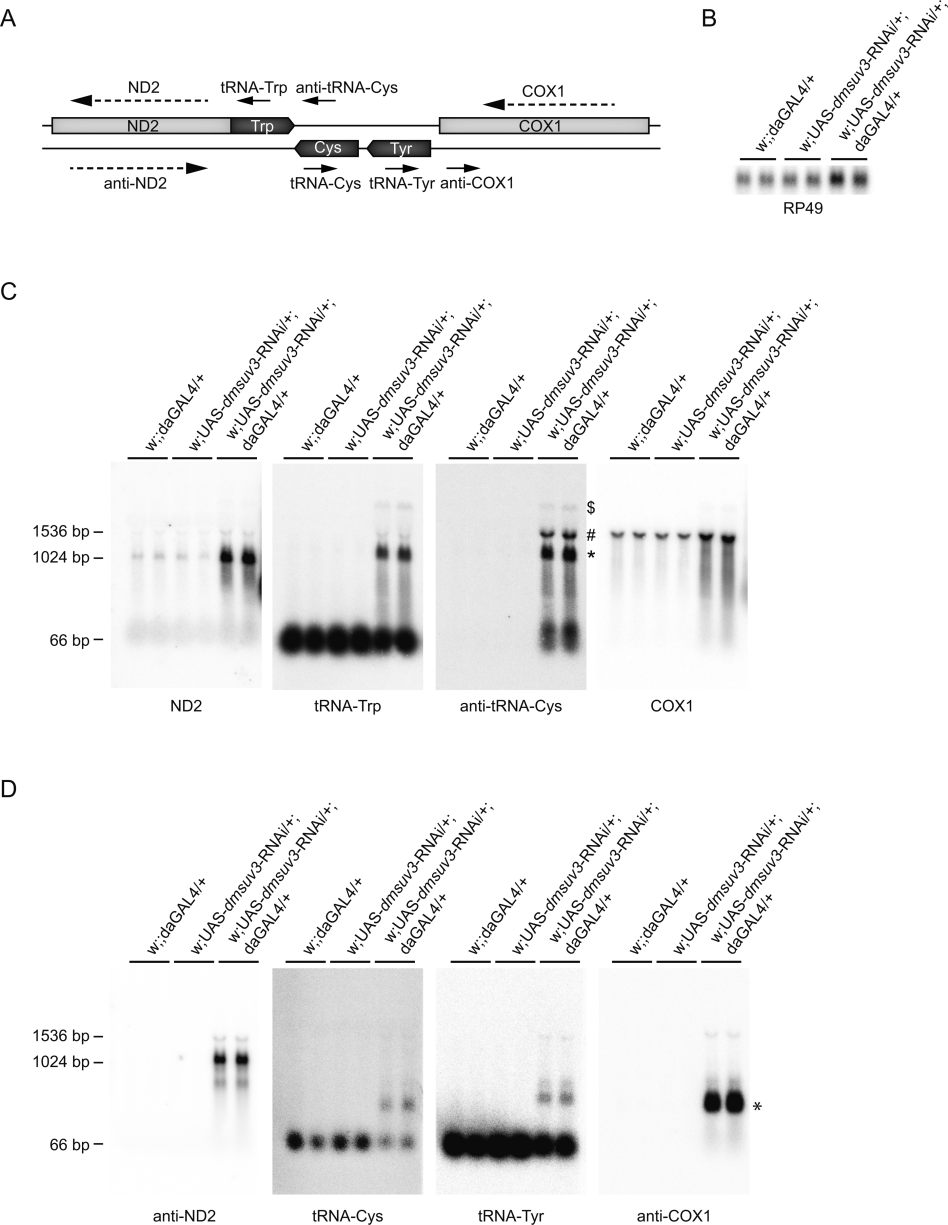
Loss of DmSUV3 leads to altered processing of the mitochondrial transcripts. (**A**) Schematic representation of the end-labeled oligonucleotide probes (black arrows) and single-stranded RNA probes (dashed arrows) used in northern blot experiments. (**B**) Northern blot analysis of the transcript encoding the nuclear ribosomal protein 49, used as a loading control. (**C** and **D**) Northern blot experiments on formaldehyde agarose separated total RNA using oligonucleotide probes and single-stranded probes, detailed in (**A**), in *dmsuv3* KD (w;UAS-*dmsuv3*-RNAi/+;daGAL4/+) and control (w;UAS-*dmsuv3*-RNAi/+; and w;;daGAL4/+) larvae at 5 days ael. * in panel (**C**) transcript containing ND2-tRNA^Trp^-anti-tRNA^Cys^, ^#^transcript containing tRNA^Trp^-anti-tRNA^Cys^-COX1, ^$^transcript containing ND2-tRNA^Trp^-anti-tRNA^Cys^-COX1, * in panel (**D**) processing intermediate of anti-COX1 mRNA.

High-resolution northern blot analysis revealed the accumulation of precursor transcripts containing tRNAs and non-coding flanking regions. However, strand-specific qRT-PCR also showed a significant increase in tRNA–mRNA junctions (ND2-tRNA^Trp^, anti-tRNA^Tyr^-COX1, Figure 5D). We therefore performed northern blot analysis against both mRNA and tRNA transcripts, using strand-specific or end-labeled oligonucleotide probes (Figure 6A). The strong increase of ND2 transcript in KD larvae was confirmed, using a strand-specific ribonuclotide probe (Figure 6C). Using labelled oligonucleotides against the immediately downstream tRNA^Trp^ revealed an additional band besides tRNA^Trp^ migrating at a similar size to ND2, while a labeled oligo against the non-coding region of anti-tRNA^Cys^ presented the same band (Figure 6C, asterisk). Interestingly, the anti-tRNA^Cys^ probe additionally revealed a transcript corresponding to the downstream COX1 transcript (Figure 6C, hashtag). Overexposure of the gels shows the polycistronic transcript covering at least ND2 to COX1 in KD larvae (Figure 6C, Supplementary Figure S5A, dollar) and in agreement, the transcripts containing anti-tRNA^Cys^-anti-tRNA^Tyr^-COX1 and ND2-tRNA^Trp^-anti-tRNA^Cys^-anti-tRNA^Tyr^-COX1 were found significantly increased using strand specific qRT-PCR (Supplementary Figure S5B).

We further investigated transcripts on the complement strand, using the same membrane (Figure 6D). A strand-specific probe against anti-ND2 revealed a stabilized transcript slightly bigger than ND2, which probably covers the entire anti-ND2 transcript, plus the mirror tRNAs of methionine and tryptophan on either side of ND2. In addition, we observed the accumulation of precursor transcripts containing tRNA^Cys^, tRNA^Tyr^ and a stabilized transcript encoding parts of the anti-cox1 mRNA, suggesting an unknown processing intermediate (Figure 6D, asterisk).

In accordance with these results, tRNA^Phe^ is also found in unprocessed precursor RNA molecules containing ND5 (Supplementary Figure S5C and S5D), and the tRNA^Phe^–ND5 junction also accumulated in KD larvae using strand-specific qRT-PCR (Supplementary Figure S5E). In contrast, we did not observe accumulation of processing intermediates containing tRNA^Val^ and junction levels between tRNA^Val^-16S rRNA were not increased (Supplementary Figure S5C, S5D and S5F). Thus, our results suggest that SUV3 helicase activity is required for the correct processing of mitochondrial transcripts, and its loss leads to the accumulation of unprocessed tRNA intermediates, affecting mitochondrial translation and OXPHOS function in metazoan.

### Loss of DmPNPase does not affect processing or tRNA levels

**Figure 7.**
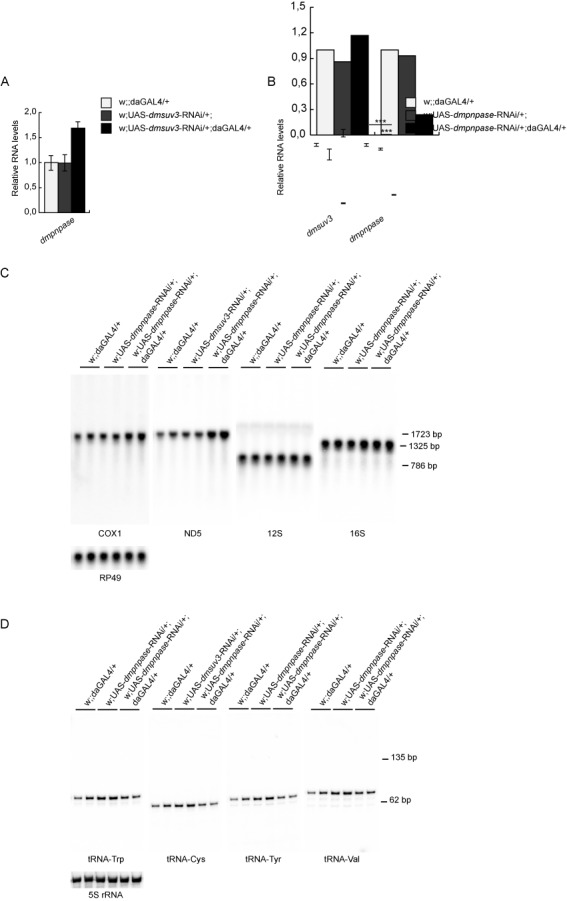
Loss of DmPNPase affects mRNA, but not tRNA levels. (**A**) qRT-PCR of *anddmpnpase* transcript in *dmsuv3* KD (w;UAS-*dmsuv3*-RNAi/+;daGAL4/+) and control (w;UAS-*dmsuv3*-RNAi/+; and w;;daGAL4/+) larvae at 5 days ael. RP49 transcript was used as endogenous control. (**B**) qRT-PCR of *dmsuv3* and *dmpnpase* transcripts in *dmpnpase* KD (w;UAS-*dmpnpase*-RNAi/+;daGAL4/+) and control (w;UAS-*dmpnpase*-RNAi/+; and w;;daGAL4/+) larvae at 4 days ael. RP49 transcript was used as endogenous control. All data are represented as mean ± SEM. (***P < 0.001, *n* = 2). (**C**) Northern blot analysis of mitochondrial rRNAs and mRNAs of *dmpnpase* KD (w;UAS-*dmpnpase*-RNAi/+;daGAL4/+) and control (w;UAS-*pnpase*-RNAi/+; and w;;daGAL4/+) larvae at 4 days ael. Loading of gels was normalised to the nuclear RP49 transcript. (**D**) Northern blot analysis of mitochondrial tRNAs of *dmpnpase* KD and control larvae at 4 days ael. Loading of gels was normalised to 5S rRNA.

To exclude that the observed processing defects are caused by the disruption of the mitochondrial degradosome, we silenced DmPNPase in flies. Similar to *Dmsuv3* silencing, *Dmpnpase* knockdown flies were pupal lethal. Interestingly, while the knockdown of DmSUV3 lead to increased DmPNPase mRNA levels, DmSUV3 expression did not change in response to DmPNPase silencing (Figure 7A and B). Although we observed increased mRNA and unchanged 12S and 16S rRNA steady-state levels (Figure 7C), comparable to observations in the DmSUV3 KD, we failed to detect processing intermediates in *dmpnpase* larvae, nor did we detect reduced tRNA levels (Figure 7D).

## DISCUSSION

SUV3 has been proposed to be an essential component of the mitochondrial degradosome in yeast (22) and humans (45). Conflicting reports on SUV3 function and the mitochondrial degradosome led us to investigate the role of the *Drosophila* homolog of SUV3. We report that DmSUV3 co-localizes exclusively with mitochondria and is required for the correct processing of mitochondrial tRNAs.

GFP-tagged DmSUV3 localized to mitochondria in transfected human tissue culture cells as well as *Drosophila* Schneider 2R+ cells, with no detection in the nucleus. Mammalian SUV3 has additionally been proposed to function in the nucleus, affecting the cell cycle, apoptosis, recombination and chromatin maintenance pathways (46–48). Although we cannot exclude that DmSUV3 might have some additional nuclear function in *Dm*, DmSUV3 shows clear mitochondrial localization and its ubiquitous knock-down results in a severe mitochondrial dysfunction with decreased respiratory chain enzyme activities and complex assembly. The severe phenotype in the homozygous P-element larvae prevented us from performing a detailed biochemical investigation and we therefore focused on RNAi-induced ubiquitous silencing of DmSUV3. Our results demonstrate that DmSUV3 is essential for fly development and its loss results in larva to pupal lethality with flies unable to undergo metamorphosis.

SUV3 has been proposed to be part of the mitochondrial degradosome, interacting either with the yeast Dss1 (22) or mammalian PNPase (24) ribonucleases to degrade mitochondrial RNAs. In agreement, silencing of both DmSUV3 and DmPNPase resulted in increased mitochondrial mRNA stability, and *de novo* transcription was not increased in DmSUV3 KD larva. In contrast, rRNA levels did not increase, suggesting that PNPase and SUV3 are not involved in a general mitochondrial RNA degradosome. PNPase has been suggested to be involved in the polyadenylation-dependent degradation of mitochondrial RNAs (7), although both mature rRNA transcripts are polyadenylated in the fly (41,49,50), questioning such a role. Further, we observed only a mild increase in degradation intermediates, questioning a causative role in the observed mitochondrial defect and larva lethality.

The tRNA punctuation model requires the tRNAs to adapt a conformation recognized by the endonuclease complexes RNase P and Z, allowing their release from the polycistronic primary transcripts (3,51). In *Dm*, 5′ processing of tRNA junctions is suggested to occur prior to 3′ processing (52), although clusters of tRNAs are suggested to be processed from the downstream end (41). Silencing of *dmsuv3* resulted in a significant reduction of numerous tRNAs, which were found accumulated in unprocessed precursors containing both protein-coding and non-coding transcripts (summarised in Table S2). Mitochondrial RNA maturation of canonical transcripts occurs via an endonucleolytic attack of RNase P and Z, and thus, these intermediates are unlikely degradation intermediates.

It is therefore likely that DmSUV3 is important for processing of primary transcripts. Failure to correctly release the tRNAs from these transcripts might therefore not only lead to tRNA depletion, as observed here, but also interfere with translation. Severe tRNA depletion has also been observed in human cell lines expressing a dominant-negative mutant of SUPV3L1, although no explanation was given (23). The importance of SUV3 helicase activity in the processing of primary transcripts has previously been suggested in both budding (21,53) and fission (54) yeast, and thus might be a major function of SUV3 in Metazoans. Support stems from a recent observation in a screen for mitochondrial proteins involved in mitochondrial RNA processing, where SUPV3L1 clustered together with components of the RNase P and Z processing machinery and SUPV3L knockdown by RNAi in a human cell line resulted in increased transcript junctions of the mitochondrial rRNAs (25). Additionally, RNase P and RNase Z were proposed to form a stable supercomplex with the yeast degradosome (55), further supporting the possibility that SUV3 helicase activity is required to resolve structures that can interfere with correct processing. SUV3 and PNPase activity has recently been proposed for the maturation of human ND6 (56), and the accumulation of unprocessed polycistronic transcripts spanning several transcripts, in KD larvae further supports SUV3 involvement in transcript maturation. Some of these polycistronic transcripts contained non-coding regions, or represented short processing intermediates, such as anti-COX1 and tRNA^Tyr^. It is thus tempting to suggest that correct processing from coding regions is required for the rapid degradation of non-coding transcripts.

It is generally accepted that poly(A) tail length in mammalian and arthropod mitochondria consists of up to 50 adenines, posttranscriptionally added to the immature RNA (10). In human (7) and mouse (8) ND6 is an exception, lacking a poly(A) tail, while it is polyadenylated in flies (33,41). Recently it has been proposed that SUV3 is involved in the deadenylation of mtRNAs in human cell culture, as the overexpression of a dominant negative mutant of hSUPVL3 resulted in an increase in poly(A) tail length (23). In contrast, recent *in vitro* data demonstrated a conditional interaction between SUV3 with both PNPase, and the mitochondrial poly(A) polymerase (mtPAP) under low mitochondrial matrix P_i_ conditions, promoting the polymerase function of mtPAP (57). This latter observation is in agreement with our results, where silencing of SUV3 in flies leads to a reduction of poly(A) tails of several transcripts, including ND1, ND3, ND6, ATP6/8 and 16S rRNA. Decreased polyadenylation has previously been shown due to the loss of LRPPRC in mice (8) as well as its fly homolog, BSF (33). LRPPRC/BSF is proposed to be involved in promoting mRNA stability in humans (58), mice (8) and flies (33), as well as being required for polyadenylation and maturation of mitochondrial mRNAs. On the other hand, silencing of DmSUV3 results in decreased poly(A) tail length, but increased mRNA steady-state levels. Severe reduction of poly(A) tails has been reported in patients with spastic ataxia with optic atrophy due to mutations in mtPAP (11), resulting in both increased and decreased mRNA steady-state levels (59). A similar trend was observed in human cell lines with silenced mtPAP (26,60), suggesting that polyadenylation does not universally regulate the stability of mitochondrial mRNAs. Interestingly, targeting of a cytosolic poly(A)-specific ribonuclease to mitochondria results in a marked translational defect (61). We also observed a marked decrease in *de novo* translation and complex assembly, consistent with the poly(A) tail being important for the regulation of mitochondrial translation. In contrast, silencing of BSF resulted in increased *de novo* translation, despite reduced polyadenylation (33), further complicating the role of the poly(A) tail in mitochondria. Thus, the role of polyadenylation of mitochondrial RNAs remains a conundrum, with its only established role to resolve incomplete termination signals in the majority of mRNAs and decreased poly(A) tail length might only be a secondary event due DmSUV3 KD.

Experiments in human (45), mouse (62) and yeast (21) associated the silencing or loss of SUV3 with a severe mtDNA depletion, suggesting a function of SUV3 during mtDNA replication. In the mouse, heterozygous disruption of SUV3 resulted in mtDNA depletion and the accumulation of mtDNA mutations, leading to tumor development and shortened lifespan (62). In contrast, we observed no changes in mtDNA steady-state levels in P-element larvae and a marked increase in mtDNA steady-state levels in KD larvae, most likely due to a compensatory effect of the mitochondrial disruption, previously observed both in flies and mouse. In mammals two origins of replication are known, with one origin placed within an ∼1kb non-coding regulatory region containing a displacement loop, while the second is embedded in a cluster of five tRNAs, two thirds around the mitochondrial genome (2). Both origins require RNA primase activity for mtDNA replication (2,63), and it is thus possible that metazoan SUV3 is necessary to release unprocessed RNAs from the promoters to allow DNA replication. Support stems from the observation that the petite-negative phenotype in yeast SUV3 mutants can be rescued in intronless yeast strains (64). However, further work is required to identify the role of SUV3 in mtDNA replication, in mammals as in the fly.

## Supplementary Material

SUPPLEMENTARY DATA
