# Supplementary material for: SUV3 helicase is required for correct processing of mitochondrial transcripts
Source: Nucleic Acids Res. 2015 Jul 7;43(15):7398–413. doi: 10.1093/nar/gkv692 (PMC4551930; doi:10.1093/nar/gkv692)
Supplement: SUPPLEMENTARY DATA [file supp_43_15_7398__index.html]

SUV3 helicase is required for correct processing of mitochondrial transcripts — SUPPLEMENTARY DATA 

# SUV3 helicase is required for correct processing of mitochondrial transcripts

## SUPPLEMENTARY DATA

- SUPPLEMENTARY DATA
- SUPPLEMENTARY DATA
- SUPPLEMENTARY DATA
- SUPPLEMENTARY DATA
