# Supplementary material for: SUV3 helicase is required for correct processing of mitochondrial transcripts
Source: Nucleic Acids Res. 2015 Jul 7;43(15):7398–413. doi: 10.1093/nar/gkv692 (PMC4551930; doi:10.1093/nar/gkv692)
Supplement: SUPPLEMENTARY DATA [file supp_gkv692_nar-00740-f-2015-File009.docx]

| **Taqman Assays** | |  |  |
| --- | --- | --- | --- |
| DmSUV3 | Dm02136650_g1 |  |  |
| DmRpL32 | Dm02151827_g1 |  |  |
| DmPNPase | Dm02362013_s1 |  |  |
| **Primers for mutagenesis and cloning of DmSUV3 in pEGFP** | |  |  |
| DmSUV3_M1 | CACTCCCTGCGATCTGGTCACAGGCGAGGAGCG |  |  |
| DmSUV3_M2 | CGCTCCTCGCCTGTGACCAGATCGCAGGGAGTG |  |  |
| DmSUV3_F | ATCTCGAGATGCAAAACTGCAGGCGCTG |  |  |
| DmSUV3_R | ATCCGCGGATTTTCTGCGTTTTTTCCTTGTCTTTC |  |  |
| **Primers for qPCR quantification of mtDNA** | |  |  |
| 16S_F | ACCTGGCTTACACCGGTTT |  |  |
| 16S_R | GGGTGTAGCCGTTCAAATTT |  |  |
| COX3_F | CAGACTCAATTTATGGATCAACATT |  |  |
| COX3_R | AAAGTTGTTCCGATTAATACATGAA |  |  |
| RP49_F | CGGATCGATATGCTAAGCTGT |  |  |
| RP49_R | CGACGCACTCTGTTGTCG |  |  |
| **Northern blot oligonucleotide probes** | |  |  |
| tRNA-Gln | AACTTTTGTGCATCATACACCAA |  |  |
| tRNA-Trp | CTTTATTTATAGCTTTGAAGGTTATTAG |  |  |
| tRNA-Cys | GCCTTAGTAAAACTTACTCCTTCAAAA |  |  |
| tRNA-Tyr | ATCTATCGCCTAAACTTCAGCC |  |  |
| tRNA-Gly | AATAGACCTTATGATTGGAAGTCAA |  |  |
| tRNA-Ala | AATGTTATATTTAACTACAACCCT |  |  |
| tRNA-Asn | TCATTAACAGTGATATGCCTC |  |  |
| tRNA-Phe | CTTCAATGTCAAACTCTAGT |  |  |
| tRNA-Thr | TTTTTGATTTACAAGACCAATGTTTT |  |  |
| tRNA-Pro | AGAAATTTCTTTTTCATTAATCCCCAAA |  |  |
| tRNA-Val | TTTGCACAAAAATCTTTTCAATG |  |  |
| anti-tRNA-Ile | CAGTTTTCTGCATTCATTGACTGATT |  |  |
| anti-tRNA-Met | CTAATTAAGCTACTGGGTTCATACCC |  |  |
| anti-tRNA-Trp | CTAATAACCTTCAAAGCTATAAATAAAG |  |  |
| anti-tRNA-Cys | TTTTGAAGGAGTAAGTTTTACTAAGGC |  |  |
| anti-COX1 | ATCGCGACAATGATTATTTTCTACAA |  |  |
| ND2 | AGAAATTAAAAATAATCCAAAAATTGAAAAAAAAGTTATA |  |  |
| **Primers for RT-PCR and circularization of mitochondrial mRNAs** | |  |  |
| 16S_R | TAATAAACACTGATACACAAGGTACAA |  |  |
| 16S_F | GACCTCGATGTTGGATTAAGATATAAT |  |  |
| ATP6/8_R | ATAAATAATAATAATCATCTAATA |  |  |
| ATP6/8_F | AATCTTATGTGTTTGCTGTAT |  |  |
| ND1_R | AGATAATAAAGGATAAGTT |  |  |
| ND1_F | GGAACTTTACCTCGATTT |  |  |
| ND3_R | GATCTTTTTTCTCGGTCGATT |  |  |
| ND3_F | GAGATTGCATTAATTCTACCT |  |  |
| ND6_R | TGTTTGAATTAATAAAGTTAATCCT |  |  |
| ND6_F | TAATGATATACAATCTATTATTAATA |  |  |
| **Primers for qRT-PCR of tRNA-mRNA junctions** | |  |  |
| tRNA-Ile_H48 | AGTTTTCTGCATTCATTGACTGA |  |  |
| tRNA-Gln_H137 | AACTTTTGTGCATCATACACCAA |  |  |
| tRNA-Gln_L158 | TGGTGTATGATGCACAAAAGTTT |  |  |
| tRNA-Met_H182 | CTAATTAAGCTACTGGGTTCATACCC |  |  |
| tRNA-Met_L221 | AACCTTTATAAATGGGGTATGAACC |  |  |
| ND2_H1217 | TTCAATTTTTGGATTATTTTTAATTTCT |  |  |
| tRNA-Trp_H1284 | CTAATAACCTTCAAAGCTATAAATAAAG |  |  |
| tRNA-Trp_L1311 | CTTTATTTATAGCTTTGAAGGTTATTAG |  |  |
| tRNA-Cys_H1324 | GCCTTAGTAAAACTTACTCCTTCAAAA |  |  |
| tRNA-Cys_L1350 | TTTTGAAGGAGTAAGTTTTACTAAGGC |  |  |
| tRNA-Tyr_H1439 | ATCTATCGCCTAAACTTCAGCC |  |  |
| tRNA-Tyr_L1459 | GCTGAAGTTTAGGCGATAGATTG |  |  |
| COX1_L1499 | TTTGTAGAAAATAATCATTGTCGCG |  |  |
| COX3_H5499 | TCACAATTTACTGATGAGGAGGA |  |  |
| tRNA-Gly_H5564 | ATTTGACTTCCAATCATAAGGTC |  |  |
| tRNA-Gly_L5590 | AATAGACCTTATGATTGGAAGTCAA |  |  |
| ND3_L5655 | AGTTGTAATTAGTAAAATTAATAAAGCA |  |  |
| tRNA-Phe_6375 | TCAAACTCTAGTATAAGCTATTTGGA |  |  |
| ND5_L6534 | GGAGGTCAACATTTATATCAAAAA |  |  |
| ND4L_H9761 | CGATTAGAAACAAAACAAAATAGCCC |  |  |
| tRNA-Thr_L9878 | TTTTGATTTACAAGACCAATGTT |  |  |
| tRNA-Thr_H9846 | GTTTAATAAAAACATTGGTCTTGT |  |  |
| tRNA-Pro_L9959 | AGTTTATTTAAAATATTAATTTTGGGGA |  |  |
| 16S_H13970 | TTTCTTTAAACAATACTAAAACTTTAAA |  |  |
| tRNA-Val_L14096 | ACATTGAAAAGATTTTTGTGCAA |  |  |

**Table S1. Taqman Probes and oligonucleotides used in this study**
