# Supplementary material for: SUV3 helicase is required for correct processing of mitochondrial transcripts
Source: Nucleic Acids Res. 2015 Jul 7;43(15):7398–413. doi: 10.1093/nar/gkv692 (PMC4551930; doi:10.1093/nar/gkv692)
Supplement: SUPPLEMENTARY DATA [file supp_gkv692_nar-00740-f-2015-File010.docx]

| **tRNA** | **Strand** | **Steady-state levels** | | **Processing Defect** |  | |  | |
| --- | --- | --- | --- | --- | --- | --- | --- | --- |
| tRNA-Ile | Lagging | Undetermined | Undetermined / 3’ | |  |  | |  |
| tRNA-Gln | Leading | 🡻🡻🡻 | 3’ | |  |  | |  |
| tRNA-Met | Lagging | Undetermined | Undetermined | |  |  | |  |
| tRNA-Trp | Lagging | Undetermined | 5’ / 3’ | |  |  | |  |
| tRNA-Cys | Leading | 🡻🡻 | 5’ | |  |  | |  |
| tRNA-Tyr | Leading | 🡻🡻 | 5’ / 3’ | |  |  | |  |
| tRNA-Gly | Lagging | 🡻🡻 | Undetermined | |  |  | |  |
| tRNA-Ala | Lagging | 🡻 | Undetermined | |  |  | |  |
| tRNA-Phe | Leading | 🡻 | 5’ | |  |  | |  |
| tRNA-Thr | Lagging | 🡻🡻🡻 | 3’ | |  |  | |  |
| tRNA-Pro | Leading | 🡻🡻 | Undetermined | |  |  | |  |
| tRNA-Val | Leading | = | No | |  |  | |  |

**Table S2. Summary of the steady state levels and processing defects of the tRNAs in DmSUV3 KD larvae.** = no significant differences in tRNA steady state levels, 🡻 tRNA steady-state levels from 65-80% of the control samples, 🡻🡻 tRNA steady-state levels from 50-65% of the control samples 50-65%, 🡻🡻🡻 tRNA steady-state levels <50% of the control samples. 5’/3’ indicates the junction found accumulated in the strand-specific QPCR analysis of the DmSUV3 KD larvae. Strand nomenclature according to Jõers, P. and Jacobs, H.T. Analysis of Replication Intermediates Indicates That Drosophila melanogaster Mitochondrial DNA Replicates by a Strand-Coupled Theta Mechanism. *journals.plos.org*, **8**, e5324.
