# Supplementary material for: SUV3 helicase is required for correct processing of mitochondrial transcripts
Source: Nucleic Acids Res. 2015 Jul 7;43(15):7398–413. doi: 10.1093/nar/gkv692 (PMC4551930; doi:10.1093/nar/gkv692)
Supplement: SUPPLEMENTARY DATA [file supp_gkv692_nar-00740-f-2015-File011.docx]

**Figure S1. The mitochondrial helicase SUV3 is conserved between *Drosophila melanogaster* and humans.** (**A**) ClustalW alignment of the human protein SUPV31L (NP_003162, top) and its Dm ortholog DmSUV3 (NP_649452, bottom).

**Figure S2. Increased mRNA stability in *dmsuv3* knockdown and P-element insertion larvae.** (**A**) Q-PCR analysis of mtDNA steady-state levels in control (w;;), heterozygous P-element insertion (w;;P{EPgy2}CG9791^EY12505^/TM6B-GFP) and homozygous P-element insertion (w;;P{EPgy2}CG9791^EY12505^) larvae at 3 days ael. (**B**) qRT-PCR of mitochondrial mRNAs in control, heterozygous P-element insertion and homozygous P-element insertion larvae at 3 days ael. RP49 transcript was used as endogenous control. All data are represented as mean +/- SEM. (* p<0.05, ***p<0.001, n=5). (**C**) Northern blot analysis of the steady-state levels of mitochondrial mRNAs and rRNAs in *dmsuv3* KD (w;UAS-*dmsuv3*-RNAi/+;daGAL4/+) and control (w;UAS-*dmsuv3*-RNAi/+; and w;;daGAL4/+) larvae at 5 days ael.

**Figure S3. tRNA maturation in *dmsuv3* deficient larvae.** (**A**) Northern blot analysis of the aminoacylation status of mitochondrial tRNAs in *dmsuv3* KD (w;UAS-*dmsuv3*-RNAi/+;daGAL4/+) and control (w;UAS-*dmsuv3*-RNAi/+; and w;;daGAL4/+) larvae at 5 days ael. 5S rRNA is shown as a loading control. (**B**) Northern blot analysis of mitochondrial tRNA^Val^, tRNA^Cys^ and tRNA^Tyr^ in control (w;;), heterozygous P-element insertion (w;;P{EPgy2}CG9791^EY12505^/TM6B-GFP) and homozygous P-element insertion (w;;P{EPgy2}CG9791^EY12505^) larvae at 3 days ael. 5S rRNA was used as a loading control.

**Figure S4. Loss of DmSUV3 leads to altered processing of non-transcript flanked mitochondrial tRNAs.** (**A**) Schematic representation of the end-labeled oligonucleotide probes (black arrows) and Northern blot experiments against tRNA^Gln^ and its 5' and 3' flanking regions in control (w;UAS-*dmsuv3*-RNAi/+; and w;;daGAL4/+) and *dmsuv3* KD (w;UAS-*dmsuv3*-RNAi/+;daGAL4/+) larvae at 5 days ael. (**B**) Schematic representation of the end-labeled oligonucleotide probes and Northern blot experiments against tRNA^Val^ (left panel), tRNA^Gly^ (middle panel) and tRNA^Phe^ (right panel) in KD and control larvae at 5 days ael. (**C**) Northern blot analysis of 5S rRNA, used as a loading control. (**D**) qRT-PCR of mitochondrial tRNA^Gln^, tRNA^Ile^ and tRNA^Met^ junctions in KD and control larvae at 5 days ael. RP49 transcript was used as endogenous control. (**E**) qRT-PCR of mitochondrial tRNA^Gly^, COX3 and ND3 junctions in KD and control larvae at 5 days ael. RP49 transcript was used as endogenous control. All data are represented as mean ± SEM. (* p<0.05, ** p<0.01, *** p<0.001, n=5).

**Figure S5. Loss of DmSUV3 leads to altered processing of non-transcript flanked mitochondrial tRNAs.** (**A**) Overexposure of the Northern blot experiments shown in Figure 6C in dmsuv3 KD (w;UAS-*dmsuv3*-RNAi/+;daGAL4/+) and control (w;UAS-*dmsuv3*-RNAi/+; and w;;daGAL4/+) larvae at 5 days ael. (**B**) qRT-PCR of the mitochondrial anti-tRNA^Cys^-COX1 and ND2-COX1 containing transcripts in KD and control larvae at 5 days ael. RP49 transcript was used as endogenous control. (**C**) Schematic representation of the end-labeled oligonucleotide probes (black arrows) and single-stranded RNA probes (dashed arrows) used in Northern blot experiments. (**D**) Northern blot experiments against tRNA^Phe^ and ND5 (left panel) and tRNA^Val^ (right panel) in control and *dmsuv3* KD larvae at 5 days ael. (**E**) qRT-PCR of the mitochondrial tRNA^Phe^-ND5 junction in KD and control larvae at 5 days ael. RP49 transcript was used as endogenous control. (**F**) qRT-PCR of the mitochondrial tRNA^Val^-16S junction in KD and control larvae at 5 days ael. RP49 transcript was used as endogenous control. All data are represented as mean ± SEM. (*** p<0.001, n=5).
