# Supplementary material for: SUV3 helicase is required for correct processing of mitochondrial transcripts
Source: Nucleic Acids Res. 2015 Jul 7;43(15):7398–413. doi: 10.1093/nar/gkv692 (PMC4551930; doi:10.1093/nar/gkv692)
Supplement: SUPPLEMENTARY DATA [file supp_gkv692_nar-00740-f-2015-File012.pdf]

A

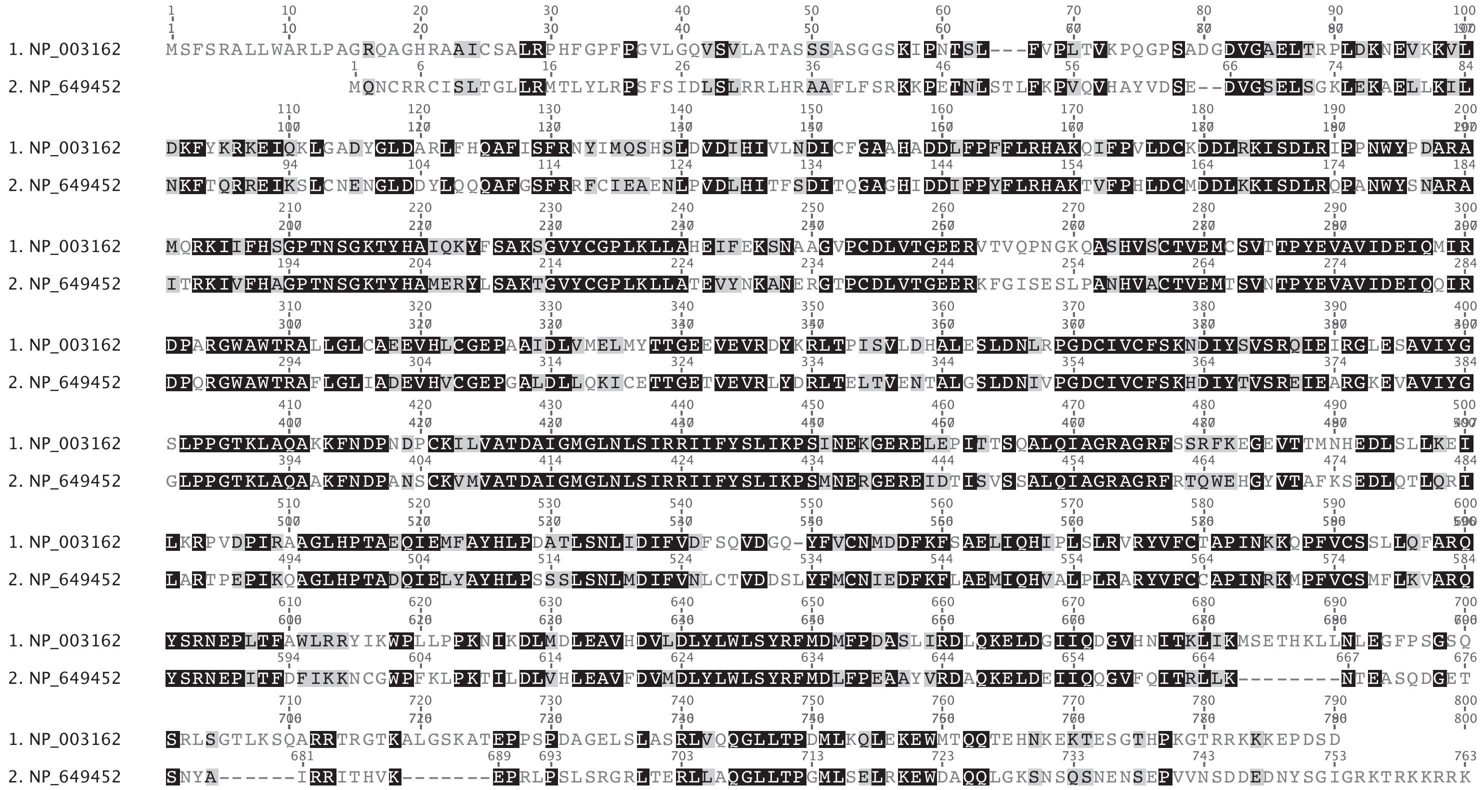

Clemente et al Figure S1

Figure S1. The mitochondrial helicase SUV3 is conserved between *Drosophila melanogaster* and humans. (**A**) ClustalW alignment of the human protein SUPV31L (NP_003162, top) and its *Dm* ortholog DmSUV3 (NP_649452, bottom).

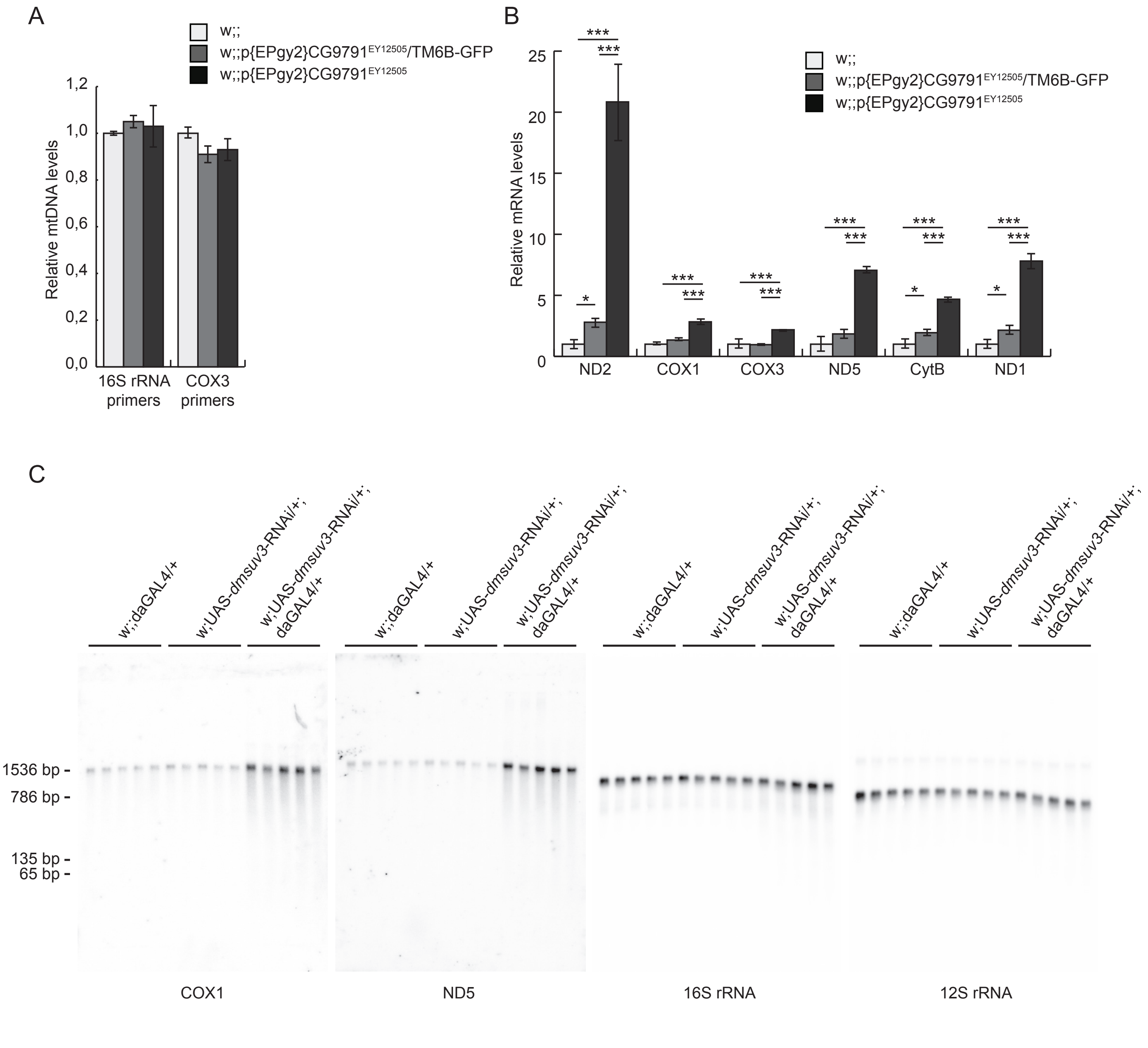

Clemente et al. Figure S2

Figure S2. Increased mRNA stability in *dmsuv3* knockdown and P-element insertion larvae. (A) Q-PCR analysis of mtDNA steady-state levels in control (w;;), heterozygous P-element insertion (w;;P{EPgy2}CG9791^{EY12505}/TM6B-GFP) and homozygous P-element insertion (w;;P{EPgy2}CG9791^{EY12505}) larvae at 3 days ael. **(B)** qRT-PCR of mitochondrial mRNAs in control, heterozygous P-element insertion and homozygous P-element insertion larvae at 3 days ael. RP49 transcript was used as endogenous control. All data are represented as mean +/- SEM. (* $p<0.05$, ***$p<0.001$, n=5). **(C)** Northern blot analysis of the steady-state levels of mitochondrial mRNAs and rRNAs in *dmsuv3* KD (w;UAS-*dmsuv3*-RNAi/+;daGAL4/+) and control (w;UAS-*dmsuv3*-RNAi/+; and w;;daGAL4/+) larvae at 5 days ael.

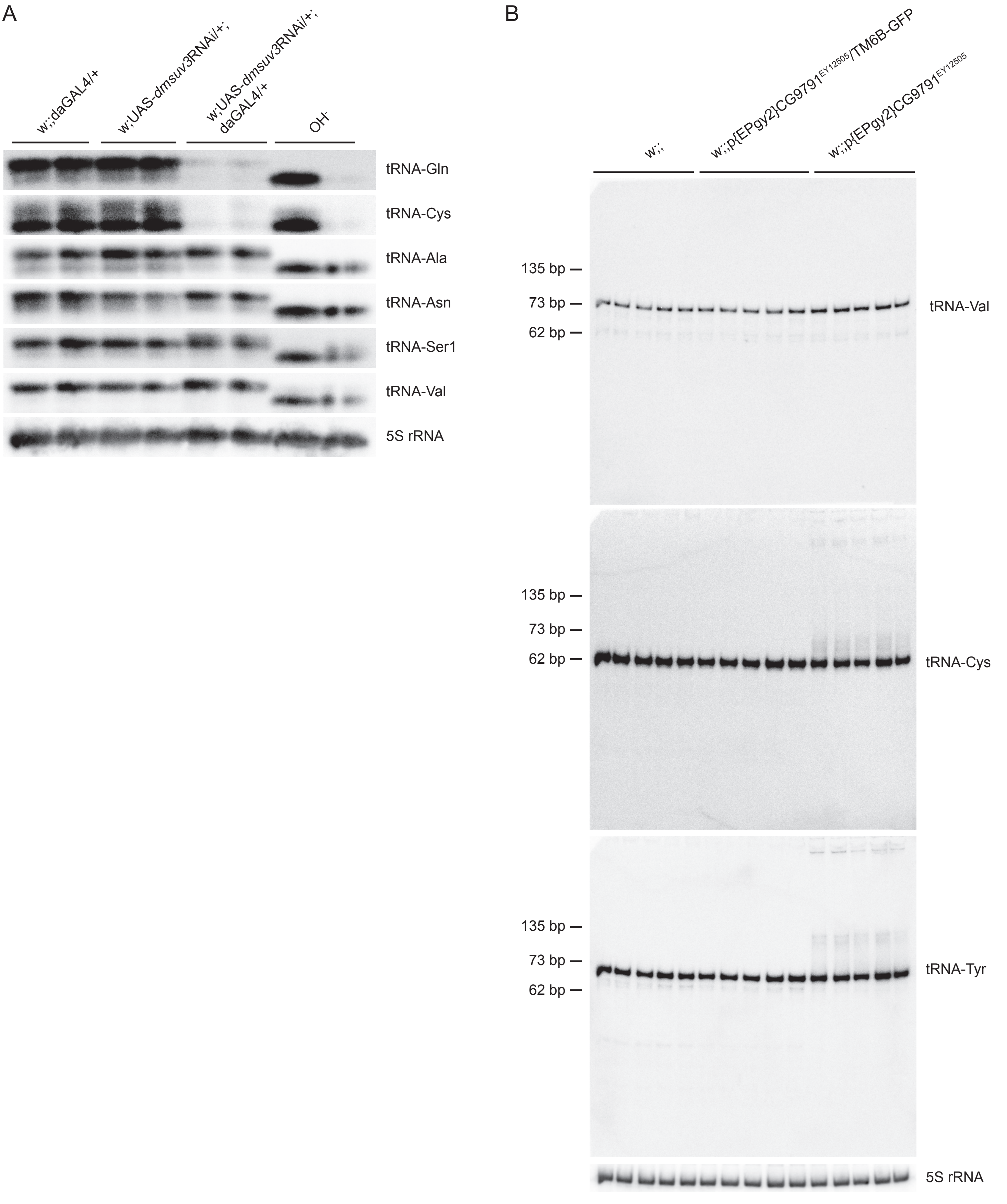

Clemente et al Figure S3

Figure S3. tRNA maturation in *dmsuv3* deficient larvae. A. Northern blot analysis of the aminoacylation status of mitochondrial tRNAs in *dmsuv3* KD (w;UAS-*dmsuv3*RNAi/+;daGAL4/+) and control (w;UAS-*dmsuv3*RNAi/+; and w;;daGAL4/+) larvae at 5 days ael. 5S rRNA is shown as a loading control **B.** Northern blot analysis of mitochondrial tRNA-Val, tRNA-Cys and tRNA-Tyr in control (w;;), heterozygous P-element insertion (w;;P{EPgy2}CG9791^{EY12505}/TM6B-GFP) and homozygous P-element insertion (w;;P{EPgy2}CG9791^{EY12505}) larvae at 3 days ael. 5S rRNA was used as a loading control.

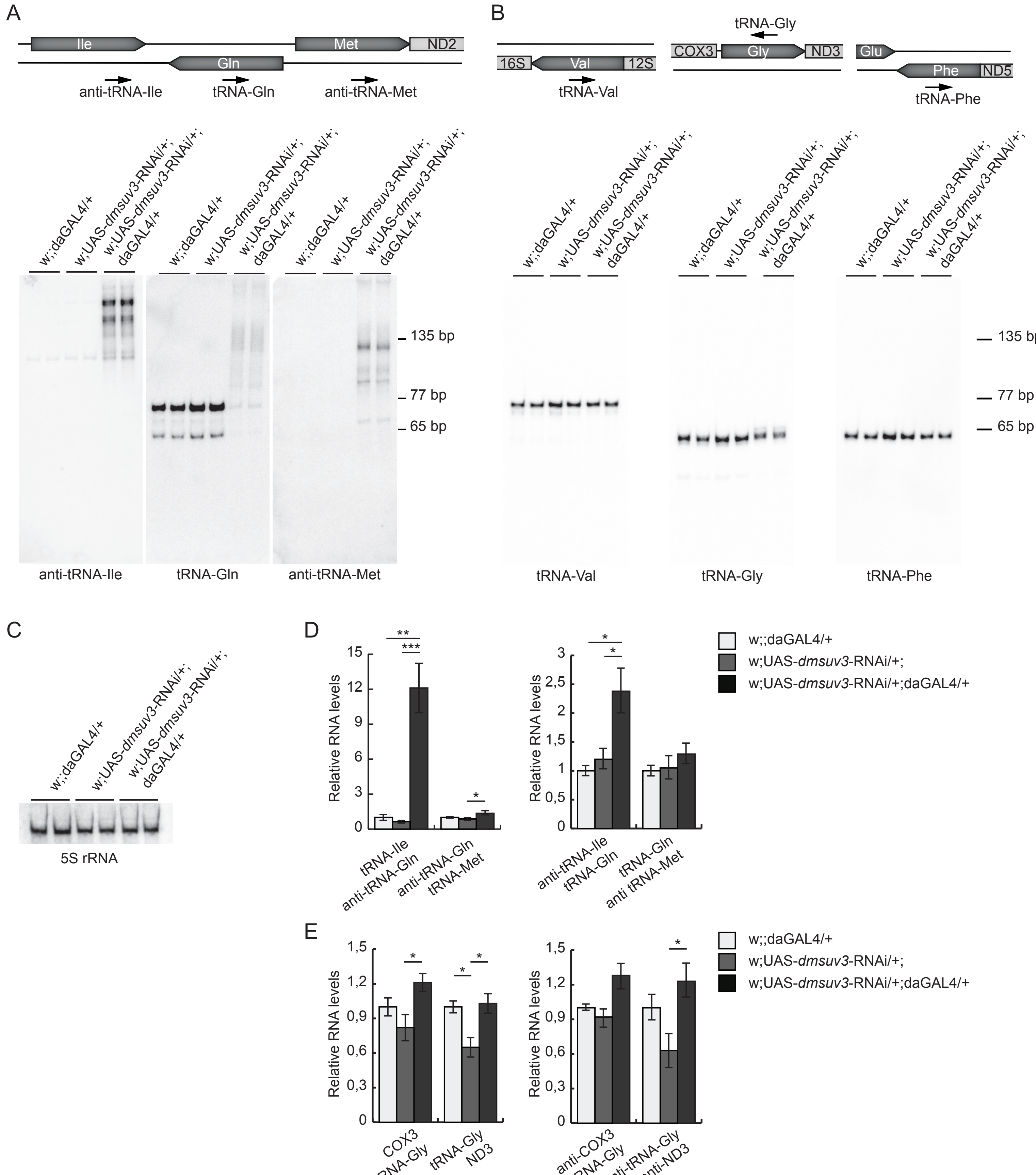

Clemente et al Figure S4

Figure S4. Loss of DmSUV3 leads to altered processing of non-transcript flanked mitochondrial tRNAs. (A) Schematic representation of the end-labeled oligonucleotide probes (black arrows) and Northern blot experiments against tRNA-Gln and its 5' and 3' flanking regions in control (w;UAS-*dmsuv3*-RNAi/+; and w;;daGAL4/+) and *dmsuv3* KD (w;UAS-*dmsuv3*-RNAi/+;daGAL4/+) larvae at 5 days ael. **(B)** Schematic representation of the end-labeled oligonucleotide probes and Northern blot experiments against tRNA-Val (left panel), tRNA-Gly (middle panel) and tRNA-Phe (right panel) in KD and control larvae at 5 days ael. **(C)** Northern blot analysis of 5S rRNA, used as a loading control. **(D)** qRT-PCR of mitochondrial tRNA-Gln, tRNA-Ile and tRNA-Met junctions in KD and control larvae at 5 days ael. RP49 transcript was used as endogenous control. **(E)** qRT-PCR of mitochondrial tRNA-Gly, COX3 and ND3 junctions in KD and control larvae at 5 days ael. RP49 transcript was used as endogenous control. All data are represented as mean ± SEM. (* $p<0.05$, ** $p<0.01$, *** $p<0.001$, n=5).

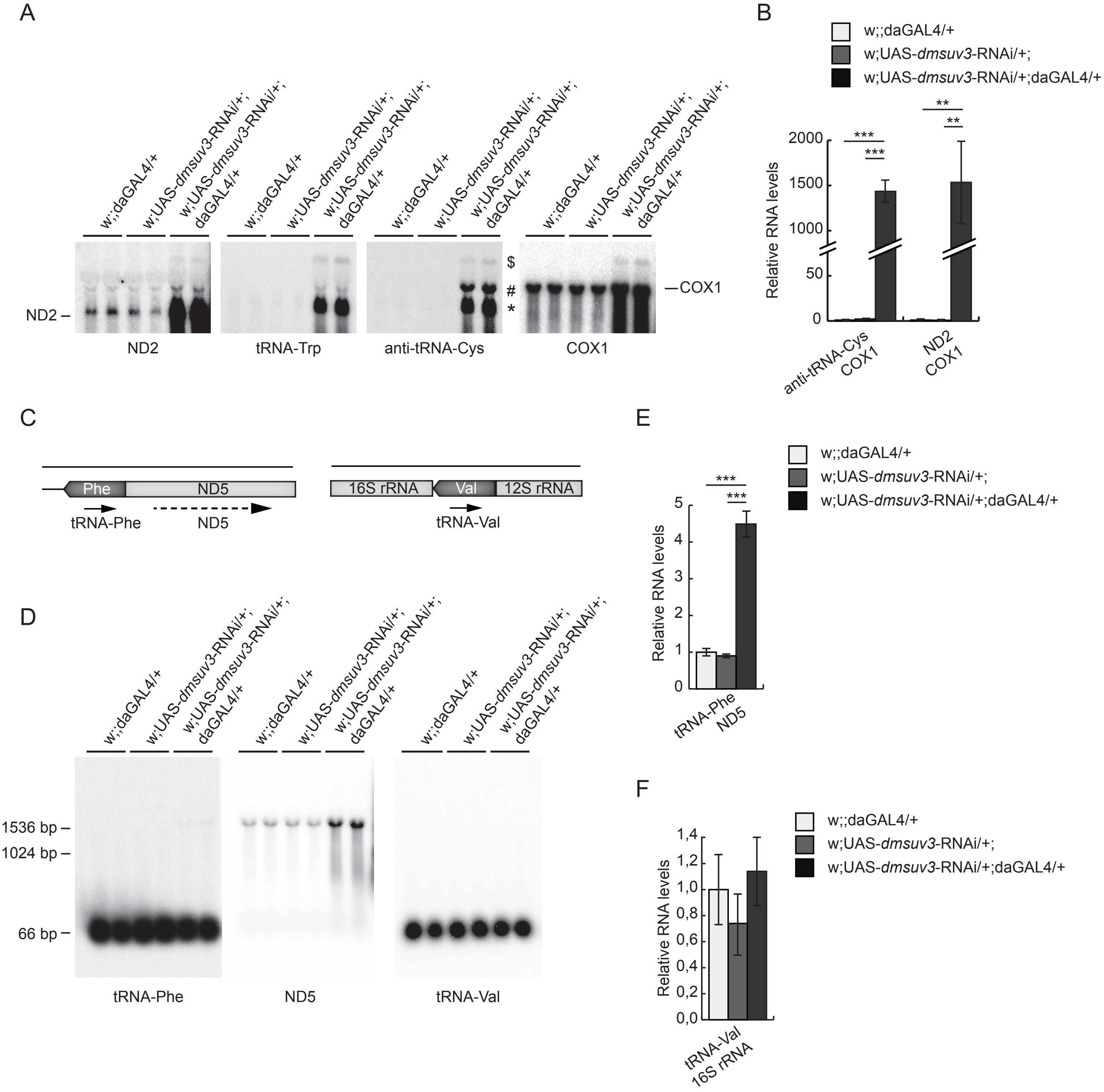

Clemente et al Figure S5

Figure S5. Loss of DmSUV3 leads to altered processing of non-transcript flanked mitochondrial tRNAs. (A) Overexposure of the Northern blot experiments shown in figure 6C in *dmsuv3* KD (w;UAS-*dmsuv3*-RNAi/+;daGAL4/+) and control (w;UAS-*dmsuv3*-RNAi/+; and w;;daGAL4/+) larvae at 5 days ael. **(B)** qRT-PCR of the mitochondrial anti-tRNA-Cys-COX1 and ND2-COX1 containing transcripts in KD and control larvae at 5 days ael. **(C)** Schematic representation of the end-labeled oligonucleotide probes (black arrows) and single-stranded RNA probes (dashed arrows) used in Northern blot experiments. **(D)** Northern blot experiments against tRNA-Phe and ND5 (left panel) and tRNA-Val (right panel) in control and *dmsuv3* KD larvae at 5 days ael. **(E)** qRT-PCR of the mitochondrial tRNA-Phe-ND5 junction in KD and control larvae at 5 days ael. RP49 transcript was used as endogenous control. **(F)** qRT-PCR of the mitochondrial tRNA-Val-16S junction in KD and control larvae at 5 days ael. RP49 transcript was used as endogenous control. All data are represented as mean ± SEM. (*** p<0.001, n=5).
